# Japanese Encephalitis Virus Persistence in Porcine Tonsils Is Associated With a Weak Induction of the Innate Immune Response, an Absence of IFNγ mRNA Expression, and a Decreased Frequency of CD4^+^CD8^+^ Double-Positive T Cells

**DOI:** 10.3389/fcimb.2022.834888

**Published:** 2022-02-24

**Authors:** Valerie Redant, Herman W. Favoreel, Kai Dallmeier, Willem Van Campe, Nick De Regge

**Affiliations:** ^1^ Operational Direction Infectious Diseases in Animals, Unit of Enzootic, Vector-borne and Bee Diseases, Sciensano, Brussels, Belgium; ^2^ Laboratory of Immunology, Department of Virology, Immunology and Parasitology, Faculty of Veterinary Medicine, Ghent University, Merelbeke, Belgium; ^3^ Katholieke Universiteit (KU) Leuven Department of Microbiology & Immunology, Rega Institute, Laboratory of Virology, Molecular Vaccinology and Vaccine Discovery, Leuven, Belgium; ^4^ Experimental Animal Center, Sciensano, Machelen, Belgium; ^5^ Operational Direction Infectious Diseases in Animals, Unit of Exotic Viruses and Particular Diseases, Sciensano, Brussels, Belgium

**Keywords:** Japanese encephalitis virus (JEV), tonsils, persistence, CD4^+^CD8^+^ double-positive T cells, IFNγ, cell-mediated immune response

## Abstract

In humans, Japanese encephalitis virus (JEV) causes a devastating neurotropic disease with high mortality, whereas in pigs, the virus only causes mild symptoms. Besides tropism to the central nervous system, JEV seems to harbor a particular tropism for the tonsils in pigs. This secondary lymphoid organ appears to act as a reservoir for the virus, and we show that it is found up to 21 days post infection at high viral titers. The immune response in the tonsils was studied over time upon intradermal inoculation of pigs. Entry of the virus in the tonsils was accompanied by a significant increase in anti-viral OAS1 and IFNβ mRNA expression. This limited antiviral response was, however, not sufficient to stop JEV replication, and importantly, no IFNγ or innate inflammatory cytokine mRNA expression could be observed. Strikingly, the persistence of JEV in tonsils was also associated with a significant decreased frequency of CD4^+^CD8^+^ double-positive T lymphocytes. Furthermore, it is important to note that JEV persistence in tonsils occurred despite a strong induction of the adaptive immune response. JEV-specific antibodies were found after 6 days post infection in serum, and cell-mediated immune responses upon NS3 restimulation of PBMCs from experimentally infected pigs showed that CD4^+^CD8^+^ double-positive T cells were found to display the most prominent proliferation and IFNγ production among lymphocyte subtypes. Taken together, these results suggest that an inadequate induction of the innate immune response and the absence of an IFNγ antiviral response contribute to the persistence of JEV in the tonsils and is associated with a decrease in the frequency of CD4^+^CD8^+^ double-positive T cells.

## Introduction

As early as the 1870s, Japanese encephalitis virus (JEV) epidemics have been described about every 10 years in Japan. By 1935, JEV was first isolated from a human and named the Nakayama strain (Solomon et al., 2000). Currently, the virus is endemic in rural areas of South and Southeast Asia where it represents the main cause of viral encephalitis. JEV belongs to a group of mosquito-borne flaviviruses that also includes Dengue virus (DENV), Zika virus (ZIKV), and West Nile virus (WNV), which all cause devastating diseases in humans (Weaver and Barrett, 2004; van den Hurk et al., 2009; Morita et al., 2015). Being a mostly subclinical disease in humans, JEV can nonetheless cause severe clinical disease in about 1 out of 250 cases. Symptoms range from high fever and headache to coma and convulsions and mortality can reach up to 25%–30% of all clinical cases [World Health Organization (WHO), 2014]. On the other hand, in its natural host, the pig, JEV typically only causes mild symptoms, which range from fever to reduced appetite. JEV can however present itself as a reproductive disease in mature pigs and non-suppurative encephalitis can be observed in young piglets (Yamada et al., 2004). Whereas humans represent a dead-end host due to the low viremia observed, in pigs, JEV infection results in high viremia, which can last for 2–4 days. JEV may reach a very high natural infection rate in pig populations (up to 100%), which facilitates its spread to other hosts *via* mosquitoes, making the pig an ideal amplification host (van den Hurk et al., 2009). Further contributing to the importance of pigs as its natural host is the finding that JEV bears a clear tropism to the tonsils and that it persists till at least 25 days post infection (dpi) at these sites (Ricklin et al., 2016a; Ricklin et al., 2016b). This suggests that pigs are not only an important amplifying host of JEV but potentially also a reservoir host. Up till now, no explanation has been found how JEV can persist for such an extended period of time in the tonsils of pigs even despite the presence of neutralizing antibodies (Ricklin et al., 2016b; Redant et al., 2020). These neutralizing antibodies could already be found starting from 5 dpi and are a first sign of a developing adaptive immune response to JEV. Previous research into the adaptive cell-mediated immune response to JEV has solely focused on humans and pointed that non-structural viral proteins such as NS3 are frequently targeted by T cells (Kumar et al., 2004; Turtle et al., 2016). In the present study we aimed to further clarify the root of the persistence in the tonsils and study the cell-mediated immune responses upon exposure to JEV antigen in pigs.

## Materials and Methods

### Virus

JEV genotype 3 Nakayama strain was purchased from Public Health England, UK (National Collection of Pathogenic Viruses, NCPV strain no. TC 362). The virus was passaged once in Vero cells before use in the animal experiment. This resulted in a JEV virus stock with a titer of 10^6^ TCID_50_/ml. The virus was then passaged another two times in order to have a sufficient stock for downstream laboratory analyses.

### Animals and Experimental Design

The procedures executed in this experiment were approved by the ethical committee of Sciensano (20171024-01 and 20200602-01) and conducted in BSL3 animal facilities. In a first experiment, 35 8-week-old pigs (Belgian landrace sows crossbred with Piétrain boars) were brought into the BSL3 facilities and were allowed 1 week of adaptation to their new environment before the start of the experiment. The animals were assigned randomly into two groups, one group was inoculated intradermally with a dose of 10^5^ TCID_50_ and the second group was infected intradermally with PBS and kept as a control group. Three pigs from the control group were euthanized on the day of infection (0 dpi). At 1, 2, 3, 5, 7, 10, 14, and 21 dpi, one predefined pig of the control group and three predefined pigs of the intradermally inoculated group were euthanized. At the time of euthanasia, blood and tonsils were collected. An analysis of other organs sampled concomitantly has been published before (Redant et al., 2020). In a second animal experiment that aimed to study the cell-mediated immunity upon JEV infection, 31 9-week-old pigs were inoculated intradermally with the same dose. Since it was established from the first experiment that neutralizing antibodies (as first sign of a developing adaptive immune response) were detectable from 5 dpi, pigs were euthanized from 5 dpi on and followed each day till 14 dpi. At 0 dpi, three pigs of the control group were euthanized, and at 5, 6, 7, 8, 9, 10, and 14 dpi, one pig of the control group and three pigs of the intradermally inoculated group were euthanized.

### JEV Detection by qPCR

Tonsils were homogenized in 1 ml of PBS with 2 5-mm stainless steel beads (Qiagen) and high-speed shaking (4 min, 25 Hz) in a TissueLyser. Subsequently, RNA was extracted from the tonsils by use of the RNeasy Mini kit (Qiagen) according to the manufacturer’s instructions. A qPCR amplifying the 3’ NTR region of JEV (Yang et al., 2004) was used to detect the presence of JEV RNA in the RNA extracts of the tonsils. The AgPath-ID one-step RT-PCR kit was used (Thermo Fisher) for amplification with a final primer and probe concentration of 0.4 µM and 0.25 µM, respectively (**Table 1**). The RT-qPCRs were run on a LightCycler 480 (Roche) for 45 cycles. A standard curve was constructed by testing a 10-fold serial dilution of a JEV virus stock with a titer of 10^8.5^TCID_50_/ml in the qPCR described above, and used to convert Ct values in “equivalent” viral loads in TCID_50_/ml or TCID_50_/g.

**Table 1:** Sequences for forward and reverse primers and probes used for mRNA expression.

	Description	Primer or probe sequence (5’ – 3’)		
		Forward primer	Reverse primer	Probe
**JEV 3’NTR**	Japanese encephalitis virus	GGTGTAAGGACTAGAGGTTAGAGG	ATTCCCAGGTGTCAATATGCTGTT	CCCGTGGAAACAACATCATGCGGC
**Reference genes**				
ACTB	β-actin	AGCGCAAGTACTCCGTGTG	CGGACTCGTCGTACTCCTGCTT	TCGCTGTCCACCTTCCAGCAGATGT
GADPH	glyceraldehyde-3-phosphte-dehydrogenase	ACATGGCCTCCAAGGAGTAAGA	GATCGAGTTGGGGCTGTGACT	CCACCAACCCCAGCAAGAGCACGC
HPRT1	hypoxanthine phosphoribosyltransferase 1	GTGATAGATCCATTCCTATGACTGTAGA	TGAGAGATCATCTCCACCAATTACTT	ATCGCCCGTTGACTGGTCATTACAGTAGCT
**Targets of interest**				
IFNα	Interferon alpha	CAGGTCCAGAAGGCTCAAG	ATCCAGTCCAGTGCAGAAC	AGGTCTGCTGGAGCATCTCATGC
IFNβ	Interferon beta	AGCAGATCTTCGGCATTCTC	GTCATCCATCTGCCCATCAA	TAGCACTGGCTGGAATGAAACCGT
IFNγ	Interferon gamma	CGATCCTAAAGGACTATTTTAATGCAA	TTTTGTCACTCTCCTCTTTCCAAT	ACCTCAGATGTACCTAATGGTGGACCTCTT
TNFα	Tumor necrosis factor alpha	AACCTCAGATAAGCCCGTCG	ACCACCAGCTGGTTGTCTTT	CCAATGCCCTCCTGGCCAACG
IL1α	Interleukin-1 alpha	GTGCTCAAAACGAAGACGAACC	CATATTGCCATGCTTTTCCCAGAA	TGCTGAAGGAGCTGCCTGAGACACCC
IL1β	Interleukin-1 beta	AAGTGCTGCACCCAAAACCT	CACTGCCACGATGACAGACA	TGGGAGCATCCAGCTGCAAATCTC
IL2	Interleukin-2	TGCTGATCTCTCCAGGATGC	CCTCCAGAGCTTTGAGTTCTTCTACTA	AAGCAGGCTACAGAATTGAAACACCTT
IL6	Interleukin-6	CTGGCAGAAAACAACCTGAACC	TGATTCTCATCAAGCAGGTCTCC	TGGCAGAAAAAGACGGATGC
IL10	Interleukin-10	CGGCGCTGTCATCAATTTCTG	CCCCTCTCTTGGAGCTTGCTA	AGGCACTCTTCACCTCCTCCACGGC
IL18	Interleukin-18	CGGAAGACAATTGCATCAGC	TCAAACACGGCTTGATGTCC	TGAAAACGATGAAGACCTGGAATCGG
FOXP3	Forhead box P3	ACCGTGGACGAGTTCGAGTT	TTTCCTTGGCTTTGCAGTCA	CCCAGCAGGTGTTCCAACCCCA
CCL2	C-C chemokine ligand 2 (MCP-1)	TCACCAGCAGCAAGTGTCCT	AGTCAGGCTTCAAGGCTTCG	CAAGACCATCGCGGGCAAGGA
CCL5	C-C chemokine ligand 5 (RANTES)	AAGCCTTGAGCCTGAACTCG	GGAGCCCTGGGAGGTTTTAC	TTGTCCCAGCCAGCTTGGGAGG
CXCL9	C-X-C chemokine ligand 9	GCAGTGTTGCCTTGCTTTTG	TCCTTTGGCTGGTGTTGATG	CCTGACTCTGATTGGAGTTCAAGGAACCC
CXCL10	C-X-C chemokine ligand 10 (IP10)	GGGCCGGAGAGAATCTACCT	TCGGGATGATGAACCATCTG	TGCTCCCACACTTGCAGGGCA
CXCL11	C-X-C chemokine ligand 11	GGTGTGAAGGGCATGGCTA	GACACCTTCCCGCTTTGAAC	TGTGCTACAACTATTCAAGGCTTCCCCA
OAS1	2'-5'-oligoadenylate synthetase 1	GTTGTGAAGGGTGGCTCCTC	TGAGGAAGACGACGAGGTCA	ACCCTCAGGGGCCGATCAGATG
CASP1	Caspase 1	GCCCGAGCTTTGATTGACTC	GGGGGTCGTCTCCACAAATA	TCGGAAAGGGCCCCAGGCAT
NLRP3	NOD-, LRR- and pyrin domain-containing protein 3	AGCAGGAGAGGGAGCATGAG	GCACAGGCTCAGAGTGTTGG	TGGCCCCGTGAGTTCCCTGA

### JEV Isolation and Titration in Tissue Samples

Presence and quantification of infectious virus in the tonsils were determined as follows. First, homogenates were prepared as described above for JEV detection by qPCR. Thereafter, homogenates of tonsils were serially diluted till 10^-4^ and 100 µl of all dilutions was then added in quadruplicates to 90% confluent Vero cells grown in 96-well plates. After 2 h of incubation, the inoculum was removed, and cells were washed and replaced by fresh DMEM medium supplemented with 5% fetal bovine serum and 1% gentamycin and incubated at 37°C. After 72 h of incubation, the plates were put at −80°C and freeze-thawed. A second passage was then conducted by transferring 100 µl of supernatant of the first passage to 90% confluent Vero cells. This was incubated for 2 h at 37°C before replacing the inoculum with fresh DMEM medium. After 72 h of incubation at 37°C, supernatant was removed and the cell layer was fixed and permeabilized by the addition of methanol at −20°C for 20 min. Cells were subsequently stained with the primary flavivirus 3571 antibody (Santa Cruz), followed by incubation with an FITC-labeled secondary antibody (Thermo Fisher) to visualize JEV replication under the fluorescence microscope. Tissue infectivity was calculated by the use of the Reed-Muench method.

### cDNA Synthesis and Preamplification

cDNA synthesis was done as previously described (Verpoest et al., 2018; Redant et al., 2020). Following the manufacturer’s instructions, extracted RNA was treated with Turbo DNase (Thermo Fisher) to eliminate genomic DNA. DNase-treated RNA samples were then subsequently converted to cDNA using the M-MLV reverse transcriptase system (Life Technologies). For each reaction, a mix of 4 μl of 5 × first strand buffer, 2 μl of 0.1 M DTT, 1 μl of 10 nM dNTP mix, 0.2 μl of 10 × hexanucleotide mix, 0.5 μl of M-MLV RT, 8.3 μl of H_2_O, and 4 µl of RNA was prepared and incubated at 37°C for 45 min, followed by inactivation at 95°C for 10 min. A preamplification of the cDNA was then carried using the TaqMan PreAmp master mix (Applied Biosystems) following the manufacturer’s instructions in order to obtain more genetic material. cDNA was preamplified with primers of the selected reference genes and targets of interest (**Table 1**). The preamplification program was as follows: denaturation at 95°C for 10 min and 14 cycles of amplification 15 s at 95°C, 4 min at 60°C. Uniformity of the preamplification was verified by testing one selected tonsil sample for all reference genes and target genes before and after preamplification and PreAmp uniformity was calculated per reference gene (ACTB, GADPH, and HPRT1). Normalized Ct values were calculated for the different target genes before (cDNA) and after preamplification (PreAmp) according to the following formulas: ΔCT[cDNA] = CT[cDNA target gene] − CT[cDNA reference gene] and ΔCT[PreAmp] = CT[PreAmp target gene] − CT[PreAmp reference gene], respectively. The ΔΔCT was subsequently calculated from the difference of the two ΔCTs: ΔΔCT= ΔCT[PreAmp] − ΔCT[cDNA]. An ideal PreAmp uniformity is reached when ΔΔCT values lay between −1.5 and +1.5. The mean PreAmp uniformity values for the target genes in the tonsils related to the reference genes ACTB, GADPH, and HPRT1 were −1.50, −1.02, and −0.70, respectively.

### mRNA Expression by qPCR and Relative Quantification

Primers and probes for three reference genes (ACTB, GADPH, and HPRT1) and targets of interest [IFNα, IFNβ, IFNγ, TNFα, IL1α, IL2, IL6, IL10, IL18, CCL2 (MCP1), CCL4, CCL5 (RANTES), CXCL9, CXCL10 (IP10), CXCL11, OAS1, FOXP3, NLRP3, and Caspase-1] were designed during previous research and are shown in **Table 1** (Kumar et al., 2004; Turtle et al., 2016; Redant et al., 2020). If possible, a duplex qPCR was performed using 6-carboxyfluorescein (FAM)- and Hexachloro-fluorescein (HEX)-labeled probes. Each qPCR reaction consisted of 5 μl of preamplified sample, 10 μl of 2× FastStart TaqMan Probe master mix (Roche), 1 μl of primer/probe mix (final concentration of 0.5 µM and 0.25 μM, respectively), and 3 or 4 μl of H_2_O (duplex or monoplex reaction, respectively) with a final volume of 20 µl. The following qPCR program was run: polymerase activation and denaturation at 95°C for 10 min, followed by 45 cycles of denaturation at 95°C for 15 s and annealing/extension at 60°C for 45 s. All samples were run in duplicate on a LightCycler 480 real-time PCR system (Roche). Normalization of the samples was done in qbasePlus by the use of ACTB, GADPH, and HPRT1 as previously determined (Turtle et al., 2016). The quantification and normalization of the expression levels were based on the calculation of target *CT* values and reference gene *CT* values in qBasePlus software. The expression levels were normalized with respect to the selected reference genes and to technical and experimental errors. Relative expression quantification analysis relied on the qBasePlus method (Hellemans et al., 2007). Individual target gene levels for all animals were expressed relative to the average of the control group.

### Virus Neutralization Test

The presence of neutralizing antibodies in serum was assessed by virus neutralization tests (VNT). Sera were diluted 1:5 in DMEM, followed by twofold dilutions till 1:640 in 96-well plates in final volumes of 50 μl. One hundred TCID_50_ of the virus in 50 μl was added to the serum dilutions and incubated for 1 h at 37°C. Next, 10^4^ Vero cells in 100 μl of medium were added to the serum–virus mix and incubated for 6 days at 37°C. After incubation, the presence of a cytopathic effect was assessed under a light microscope. All sera were tested in duplicate. The neutralizing titer of a sample was determined as the highest dilution of the serum that was still capable to completely neutralize the virus.

### Isolation of Immune Cells From Tonsils and Blood

Upon collection, tonsils were cut in pieces and washed 3 times in PBS supplemented with 200 µg/ml gentamycin. Next, the tonsils were resuspended in RPMI containing 10% FBS, 1% NEAA, 1% sodium pyruvate, 1% penicillin–streptomycin, and 1 mg/ml Collagenase I (Gibco). The tonsils were left for approximately 3 h of incubation at 37°C with magnetic agitation. When sufficiently digested, single-cell suspensions were put over a cell strainer to eliminate the debris; cells were subsequently washed and frozen in culture medium with 10% DMSO until further use.

PBMCs were isolated as follows. Blood was diluted 1:1 in RPMI and was layered over histopaque 1077 (Sigma). After 20 min of centrifugation at 1800×g, PBMCs were collected at the interface of histopaque 1077 and serum. The PBMCs were washed and frozen in RPMI containing 10% FBS, 1% NEAA, 1% sodium pyruvate, and 1% penicillin–streptomycin with 10% DMSO until further use.

### Antibodies and FACS Staining

The following antibodies were used for the identification of T cells, NK cells, and γδ T cells: CD3 (BB23-8E6-8C8, BD), CD4 (74-12-4, BD), CD8 (76-2-11, BD), CD16 (G7, Bio-Rad), and TCR γδ (PGBL22A, Kingfisher). Dendritic cells (DC) and monocytes in the PBMCs and the tonsils were phenotyped and gated for with the following antibodies: CD172a (BL1H7, Bio-Rad), CD14 (MIL2, Bio-Rad), CADM1 (3E1, MBL), and CD163 (2A10/11, Bio-Rad). IgY antibody (Jackson Immunoresearch) was used for secondary labeling (Parra-Sanchez et al., 2019; Auray et al., 2020). The CD172a^+^CD163^+^ population should represent macrophages and monocyte-derived DCs (Parra-Sanchez et al., 2018). For the identification of macrophages in the tonsils, the antibody mix was supplemented with CD203a (PM18-7, Bio-Rad) and CD169 (3B11/11, Bio-Rad). A live/dead viability dye (Thermo Fisher) was included for all samples. Stainings were done as follows: cells were thawed and suspended at 1 × 10^6^ cells per well in a 96-well plate. All staining steps were left to incubate for 30 min, except for the Fc block, which was incubated for 10 min, and the live/dead staining, which was left to incubate for 20 min. Cells were run on the FACSverse and analysis was done by using the flowjo software (version 10.7.1). Fluorescence minus one (FMO) controls were used as well as matched isotypes for mouse IgG1, IgG2a, and IgG2b.

### Cell-Mediated Immunity Assays

Upon thawing, cells were counted and resuspended in RPMI at 5 × 10^5^ per well. For the cell proliferation assay, cells were stained with a cell proliferation dye (ebioscience) upon thawing according to the manufacturer’s instructions for 10 min at 37°C prior to plating in RPMI supplemented with 10% FBS, 1%NEAA, 1% sodium pyruvate, and 1% penicillin–streptomycin. After plating, cells were left to proliferate for 4 days in the presence of PMA/CaI (Sigma) as a positive control, 1 µg/ml NS3 peptide mix (pepmix, JPT peptides) dissolved in DMSO as JEV antigen, or culture medium supplemented with the same amount of DMSO as the NS3 pepmix as a negative control. After 4 days of incubation at 37°C, samples were stained with antibodies for the different T-cell subsets and NK cells and subsequently run on a FACSVerse. Analysis was done in flowjo, and samples were considered positive for proliferation when at least 1% of cells were found in the proliferation dye low gate and if this was at least double the amount of cells of non-stimulated samples (Turtle et al., 2016).

For the IFNγ production and secretion assay, cells were counted and resuspended at 5 × 10^5^ cells per well in RPMI supplemented with 10% FBS, 1%NEAA, 1% sodium pyruvate, and 1% penicillin–streptomycin. Next, the cells were stimulated for 18 h with 1 µg/ml of NS3 pepmix with the addition of IL18 at 50 ng/ml as a potentiator of IFNγ response upon restimulation (Riber et al., 2011). PMA/CaI was used as a positive control, and culture medium supplemented with DMSO at the same concentration of the pepmix was used as a negative control. Both the negative and positive control were also supplemented with 50 ng/ml IL18. For the IFNγ production assay, BFA (BD) was added after 18 h, and the cells were incubated for another 6 h before staining. The IFNγ production assay was subsequently stained with antibodies for all T-cell populations and NK cells as mentioned above. For cells of the IFNγ production assay, an additional intracellular staining was performed for IFNγ detection (P2G10, BD). Permeabilization was done by using the cytofix/cytoperm kit (BD). As for IFNγ secretion, supernatant was collected after 24 h of stimulation and secreted IFNγ was quantified using the porcine IFNγ ELISA kit (mabtech) according to the manufacturer’s instructions.

### Statistical Analysis

Statistical analysis was performed using GraphPad Prism. Kruskal–Wallis and Mann–Whitney tests were used. *p*-values <0.05 were considered to be significant.

## Results

### Viral Replication and Infectivity in Tonsils

**Figure 1:**
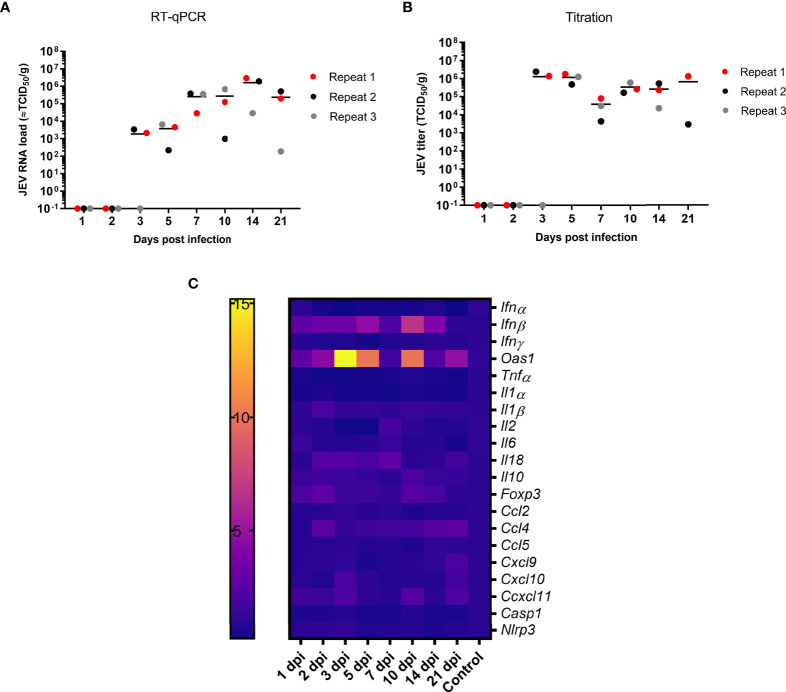
Persistence of JEV in the tonsils. **(A)** Viral load in the tonsils as determined by RT-qPCR and **(B)** amount of infectious JEV determined by virus titrations in tonsils of 9-week-old pigs upon intradermal inoculation with 10^5^ TCID_50_. Each dot represents one animal. **(C)** mRNA expression in the tonsil of intradermal inoculated 9-week-old pigs with 10^5^ TCID_50_ of JEV. Fold changes in IFNα, IFNβ, IFNγ and OAS1, TNFα, IL1α, IL2, IL6, IL10, IL18, FOXP3, CCL2, CCL5, CXCL9, CXCL10, CXCL11, NLRP3, and Caspase-1.

JEV RNA was first detected in the tonsils at 3 dpi following intradermal (i.d.) inoculation (**Figure 1A**). Upon viral manifestation in the tonsils, an active viral replication is initiated as suggested by the significant increase in JEV RNA between 3 and 14 dpi (*p* = 0.036), reaching JEV RNA levels equivalent to 10^6^ TCID_50_/g tonsil. These high amounts of viral RNA remained present in the tonsils till the end of the experiment at 21 dpi. Virus could be isolated from almost all RT-qPCR positive tonsil samples. Viral titers ranged between 10^3^ and 10^7^ TCID_50_/g. At 7 dpi, infectivity was observed at a slightly lower titer. The infectiousness in the tonsils remained high till the end of the experiment at 21 dpi (**Figure 1B**). Overall, both JEV RNA and infectious virus was found for several weeks in the tonsils of i.d. inoculated pigs, suggesting a persistent productive infection.

### Interferon and Cytokine Expression in the Tonsils

We sought to unravel the mechanism behind the persistence of JEV in the tonsils upon JEV infection. In a first step, mRNA expression of interferons (IFN), interferon-stimulated genes (ISG), and different cytokines was studied in the tonsils over time. Concomitantly with first detection of JEV RNA at 3 dpi in the tonsils, a significant, more than 15-fold upregulation of OAS1 mRNA (*p* = 0.0141) and an up to 5-fold upregulation of IFNβ mRNA (*p* = 0.0141) compared to control pigs were observed. Strikingly, the OAS1 and IFNβ mRNA expression dropped at 7 dpi to an only 2-fold increase, and increased afterwards again to a 7- and 10-fold increase of IFNβ and OAS1 mRNA at 10 dpi. IFNβ and OAS1 mRNA levels then dropped back to the levels observed in control pigs by 21 dpi (**Figure 1C**). Besides the upregulated expression of IFNβ and OAS1, no other chemokines, inflammatory cytokines, and inflammasome-related genes were found to be activated over time. Only a very minor increase in anti-inflammatory IL10 and FOXP3 mRNA could be observed at 10 dpi as well as a minor upregulation of IL2 and CCL4 mRNA at 7 dpi (**Figure 1C**). In line with the absence of induction of the innate inflammatory cytokines and chemokines, it was interesting to observe the complete absence of IFNγ mRNA upregulation (**Figure 1C**).

### Frequency of Immune Cell (Sub)Populations in the Tonsils

**Figure 2:**
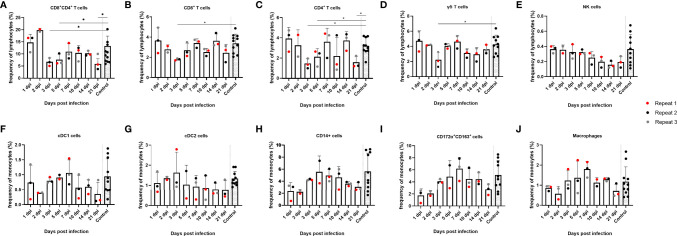
Evolution of lymphocyte and monocyte frequencies in porcine tonsils upon JEV infection. Tonsil single-cell suspensions from 9-week-old intradermally inoculated pigs with JEV were analyzed by flow cytometry. Mean frequencies of **(A)** CD4^+^CD8^+^ double-positive T cells, **(B)** CD8^+^ T cells, **(C)** CD4^+^ T cells, **(D)** γδ T cells, **(E)** NK cells, **(F)** cDC1 cells, **(G)** cDC2 cells, **(H)** CD14^+,^ **(I)** CD172a^+^CD163^+^, and **(J)** macrophages are displayed as bars. Three animals were analyzed per time point, and each dot represents one animal and expressed as the frequency of all lymphocytes for lymphocyte subsets or as the frequency of all monocytes for monocytic cell subsets. *Statistically significant different frequency compared to control animals.

Since no substantial changes in mRNA expression of most cytokines could be detected in tonsils upon JEV infection, except for IFNβ and OAS1 mRNA, we studied possible changes in frequencies of different immune cell populations in the tonsils. Cell frequency was analyzed over time for different lymphocyte and monocyte subtypes such as CD8^+^, CD4^+^, CD4^+^CD8^+^ double-positive and γδ T cells, NK cells, cDC1, cDC2, CD14^+^ monocytes, CD172a^+^CD163^+^ cells, and macrophages (**Figure 2**). Gating on lymphocytes was done as shown in **Supplementary Figure 1** and gating on monocytes was done according to Auray et al. (2020) and Parra-Sanchez et al. (2019). At 3 dpi and 5 dpi, a significant decrease in the frequency of CD4^+^CD8^+^ double-positive T cells could be observed compared to the controls (*p* = 0.011 and *p* = 0.0385, respectively), which subsequently seemed to increase again, albeit to low levels, till 7 dpi. A statistically significant decrease at 3 dpi is also observed for CD8^+^ (*p* = 0.055) and CD4^+^ (*p* = 0.0140) single-positive T cells and remained significantly decreased till 5 dpi for CD4^+^ single-positive T cells. At 7 dpi, the CD8^+^ and CD4^+^ single-positive T-cell frequencies more or less matched the frequencies that were observed at 1 and 2 dpi but seemed to decrease again at 21 dpi (**Figures 2B, C**). This decrease was statistically significant for CD4^+^ single-positive T cells compared to the control pigs (*p* = 0.140). For CD4^+^CD8^+^ double-positive T cells, however, frequencies stay well below the frequencies observed at the beginning of the experiment and significantly decreased even more by 21 dpi compared to the controls (*p* = 0.011) (**Figure 2A**). The frequency of γδ T cells over time seemed to follow the same trend as that observed for CD8^+^ and CD4^+^ single-positive T cells and was significantly decreased by 3 dpi compared to the controls (*p* = 0.0490), although the cell frequency did not decrease by 21 dpi (**Figure 2D**). The frequency of NK cells seemed to diminish by the end of the experiment, but this was not statistically significant and it has to be noted that the gating was not always straightforward because of the already low number of NK cells in the tonsils (**Figure 2E**). When analyzing the different monocyte subtypes in the tonsils, no significant differences could be detected over time. The frequency of cDC2 did not change over time (**Figure 2G**). For all other cell types, cDC1, CD14^+^ monocytes, CD172α^+^CD163^+^ cells, and macrophages, the frequency seemed to increase from 3 dpi till 7 dpi and afterwards declined to reach a steady-state level by 21 dpi (**Figures 2F, H, I, J**). Overall, the most striking finding in the tonsils is the significant reduction of CD4^+^CD8^+^ double-positive cells starting from 3 dpi.

### Humoral Immune Response

**Figure 3:**
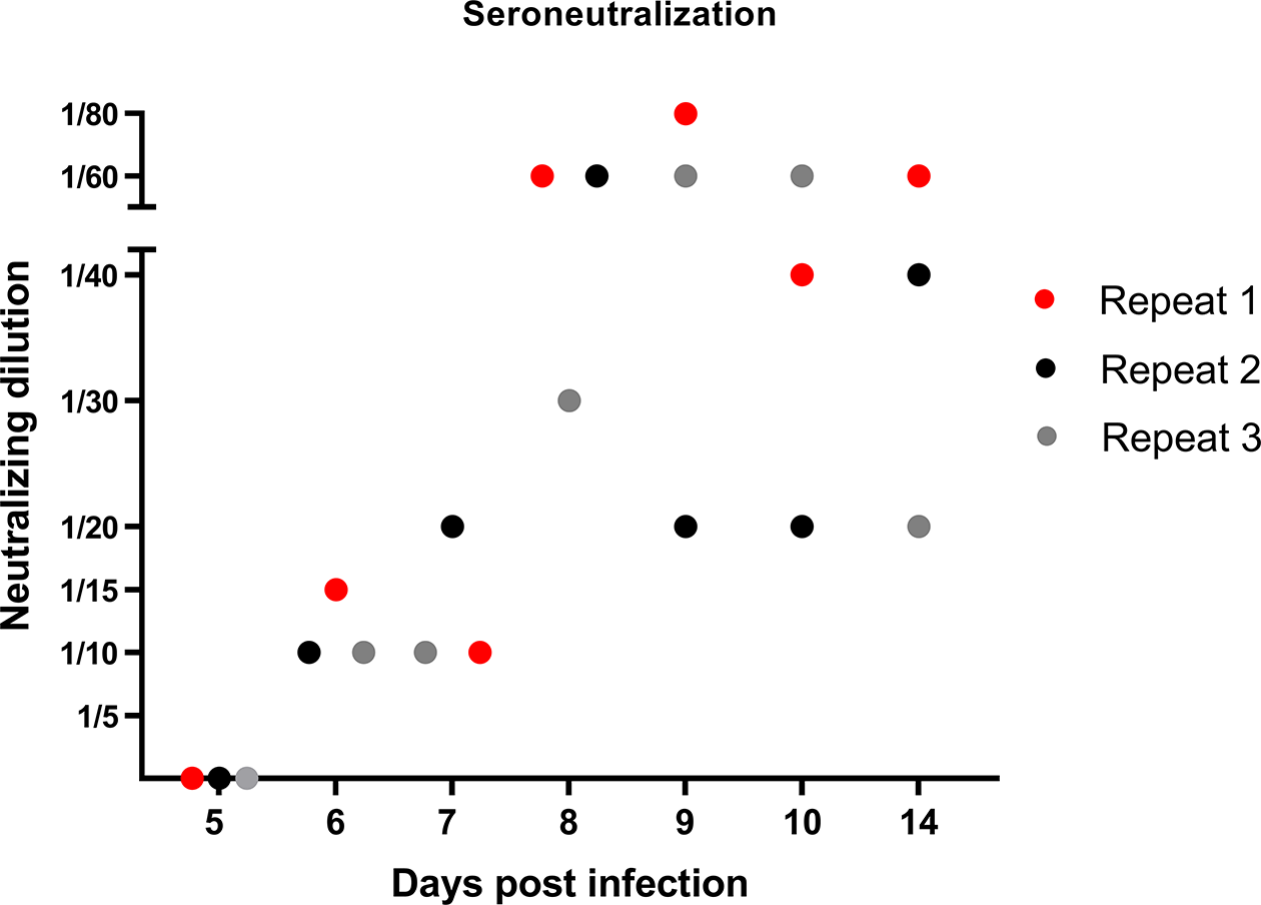
Neutralizing antibody response in JEV-infected pigs. VNT tests were performed to quantify the amount of JEV-specific neutralizing antibodies in the serum of 9-week-old pigs upon intradermal inoculation with 10^5^ TCID_50_.

After having studied the local viral replication, cytokine mRNA expression, and evolution of immune cell frequency in the tonsils, we studied the induction of the peripheral adaptive immune response upon JEV infection. In our previous publication reporting on the same pigs from the first *in vivo* experiment, we already showed that first JEV specific antibodies could be detected between 5 and 7 days post infection onwards (Redant et al., 2020). These results were confirmed in the pigs from the second experiment. Neutralizing antibodies were first detected at 6 dpi and antibody titers quickly rose the following days (**Figure 3**).

### Frequency of Immune Cell (Sub)Populations in PBMCs

**Figure 4:**
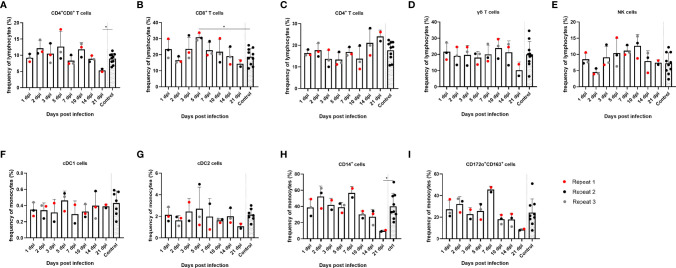
Evolution of lymphocyte and monocyte frequencies in PBMCs of JEV-infected pigs. PBMCs from 9-week-old intradermally inoculated pigs with JEV were isolated and analyzed by flow cytometry. Mean frequencies of **(A)** CD4^+^CD8^+^ double-positive T cells, **(B)** CD8^+^ T cells, **(C)** CD4^+^ T cells, **(D)** γδ T cells, **(E)** NK cells, **(F)** cDC1 cells, **(G)** cDC2 cells, **(H)** CD14^+^, and **(I)** CD172a^+^CD163^+^ are displayed as bars. Three animals were analyzed per time point, and each dot represents one animal and are expressed as the frequency of all lymphocytes for lymphocyte subsets or as the frequency of all monocytes for monocytic cell subsets. *Statistically significant different frequency compared to control animals.

After the humoral immune response, the cellular immune response in PBMCs was also studied. First, the evolution of the immune cell frequency over time was studied. With regard to lymphocytes, less significant differences were found in the frequencies of the different (sub)populations over time in PBMCs. However, a trend of reduced frequency of CD8^+^ single-positive and CD8^+^CD4^+^ double-positive T cells from 14 dpi could be noted, which was statistically significant at 21 dpi for the CD8^+^CD4^+^ double-positive T cells compared to the controls (*p* = 0.0303), as well as an increase in frequency of CD4^+^ single-positive T cells from 14 dpi. By 5 dpi, the frequency of CD8^+^ single-positive cells was temporarily significantly increased compared to the controls (*p* = 0.007) (**Figures 4A–C**). Furthermore, no significant differences were found in the frequency of NK cells and γδ T cells over time, although NK cells followed the same trend as observed for CD8^+^ single-positive and CD8^+^CD4^+^ double-positive T cells. From 14 dpi, the frequency of NK cells decreased, whereas γδ T-cell frequencies decreased at 21 dpi (**Figures 4D, E**). Next, different subtypes of the monocytic cell population were studied. Two types of conventional dendritic cells (cDC), cDC1 and cDC2, did not show significant differences over time (**Figures 4F, G**). CD14^+^ monocytes, however, did show an increase at 7 dpi and subsequently decreased in frequency starting from 10 dpi and were significantly decreased by 30% at 21 dpi compared to the controls (*p* = 0.036) (**Figure 4H**). The same trend could be observed for the CD172α^+^CD163^+^ population; however, these findings were not statistically significant for either cell type (**Figure 4I**).

### Cell-Mediated Immune Response Upon *In Vitro* NS3 Exposure

**Figure 5:**
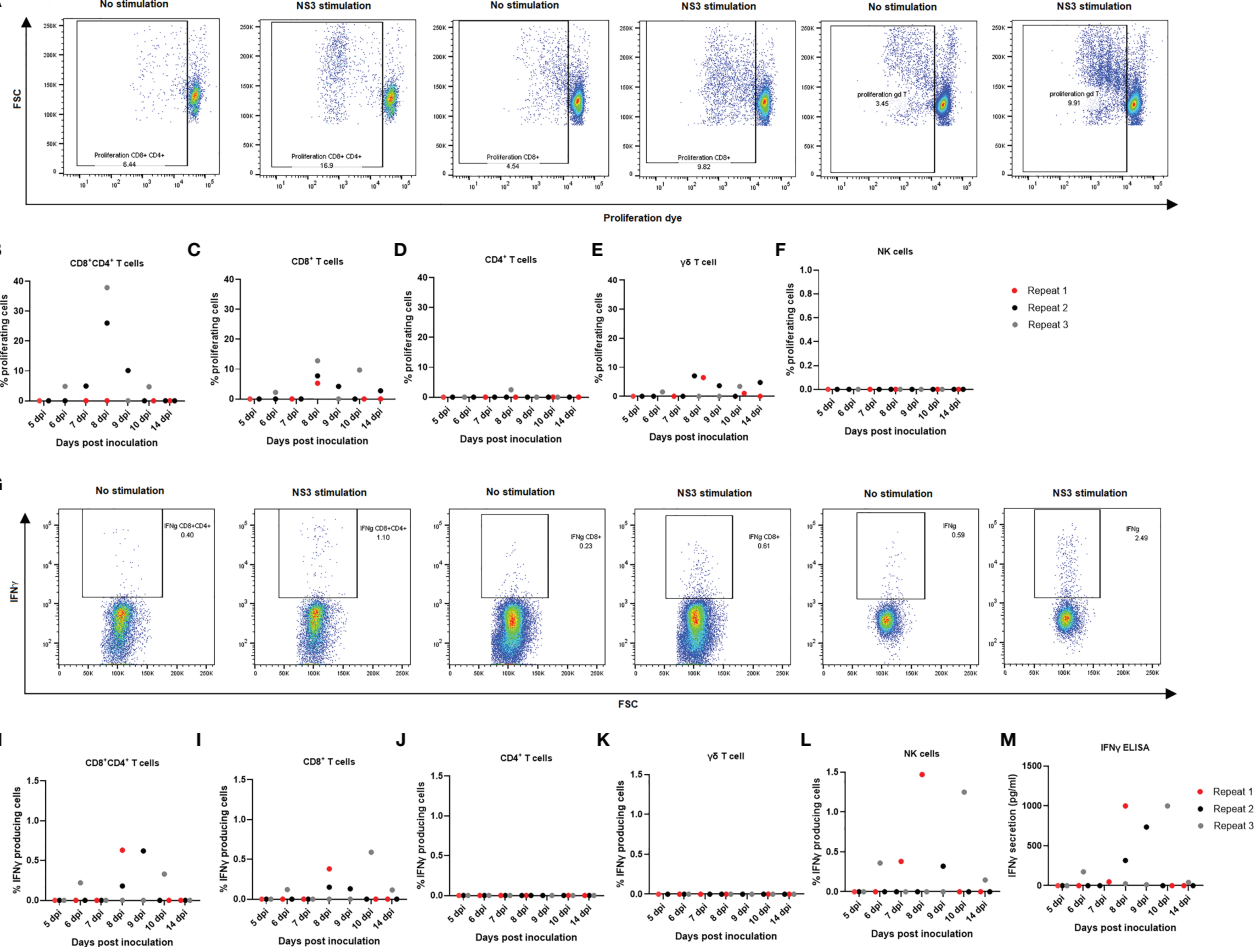
Detection of cell-mediated immune responses after *in vitro* stimulation of PBMCs from JEV-infected pigs with NS3 antigen. Isolated PBMCs from three 9-week-old pigs per time point were left to proliferate for 4 days in the presence of NS3 antigen. **(A)** Flow cytometry analysis of proliferation for **(B)** CD4^+^CD8^+^ double-positive T cells, **(C)** CD8^+^ T cells, **(D)** CD4^+^T cells, **(E)** γδ T cells, and **(F)** NK cells. Each dot represents one animal. Isolated PBMCs were incubated for 18 h in the presence of the NS3 pepmix and IL18 before adding BFA for the final 6 h. **(G)** Flow cytometry analysis of IFNγ production for **(H)** CD4^+^CD8^+^ double-positive T cells, **(I)** CD8^+^ T cells, **(J)** CD4^+^T cells, **(K)** γδ T cells, and **(L)** NK cells. One dot represents one animal. **(M)** These results were confirmed with an IFNγ ELISA after 24 h of stimulation of isolated PBMCs in the presence of NS3 antigen.

Since a quantitative decrease was noticed in overall lymphocyte populations, particularly CD8^+^ single-positive and CD4^+^CD8^+^ double-positive T lymphocytes, both in tonsils and in PBMCs, we aimed at evaluating to which extent the different cell types reacted upon restimulation with JEV antigens and from which day onwards. The cells from non-infected animals did not show any degree of proliferation upon NS3 stimulation in any of the studied cell types (**Supplementary Figure 2A**). Upon stimulation with the NS3 pepmix, mainly a CD8^+^, CD8^+^CD4^+^ double-positive, and γδ T-cell proliferation could be observed (**Figures 5A–C, E**). Proliferation was already detectable at 6 dpi, and in particular, CD4^+^CD8^+^ T cells showed the highest degree of proliferation of almost up to 40% (**Figure 5B**), followed by CD8^+^ T cells with proliferation up to almost 15% (**Figure 5C**). Strikingly, γδ T cells also showed a pronounced degree of proliferation of up to 10% of cells (**Figure 5E**). As for CD4^+^ T lymphocytes, only one of the three samples showed a modest proliferation, and no proliferation at all was observed in the NK cell population (**Figures 5D, F**). It should however be noted that only a small fraction of NK cells remained after 4 days of proliferation compared to the other cell types and compared to the normal frequency of NK cells among PBMCs or even compared to the amount of NK cells found at the end of the IFNγ assay.

Next, production of IFNγ was assessed *via* flow cytometry in CD8^+^, CD4^+^, CD4^+^CD8^+^ double-positive, γδ T cells and NK cells upon restimulation of PBMCs with NS3 peptide in combination with IL18 as co-stimulus to enhance the IFNγ signal. In the γδ T-cell population, no substantial production of IFNγ could be observed (**Figure 5K**). Also, no detectable production of IFNγ could be observed in the CD4^+^ T-cell population (**Figure 5J**). Nevertheless, starting from 6 dpi, a mainly CD8+ and CD4^+^CD8^+^ double-positive T cell-mediated and NK cell-mediated IFNγ response was observed. Analyzing the responses of the individual animals indicated that the highest response in IFNγ production was due to NK cells, followed by the CD4^+^CD8^+^ double-positive T-cell fraction and lastly the CD8^+^ T-cell population (**Figures 5G, H–L**). IFNγ production was further studied in stimulated and non-stimulated cells in all control animals, without detectable IFNγ production upon stimulation with NS3 in any of the cell subtypes studied (**Supplementary Figure 2B**). To confirm these results, an IFNγ ELISA was conducted. No IFNγ secretion could be observed in the non-infected animals upon stimulation with NS3 or in non-stimulated cells of non-infected controls and JEV-inoculated animals. IFNγ secretion was however confirmed in all animals that stained positive for IFNγ by intracellular staining in flow cytometry (**Figure 5M**).

## Discussion

Despite a short-lived viremia in pigs, JEV has been shown to persist in the porcine tonsils (Ricklin et al., 2016a; Ricklin et al., 2016b). Combined with the high viremia observed in these animals, this persistence may explain the important role pigs play as reservoir and amplification host for JEV. In the present study, *in vivo* experiments were conducted in pigs to study the immune responses occurring in the tonsils and blood over time.

High loads of infectious JEV could be found in the tonsils of the pigs till 21 dpi and likely beyond. This result is in agreement with previous studies, further supporting that tonsils constitute an important site of productive viral replication and JEV persistence (Ricklin et al., 2016a; Ricklin et al., 2016b). Previously, also other secondary lymphoid organs like prescapulary lymph nodes and spleen were studied, but no indications of productive viral replication could be found in those (Redant et al., 2020). The detection of JEV RNA in tonsils at 3 dpi and the increase of virus titers over time were associated with a significantly increased IFNβ and OAS1 mRNA expression, indicating an induction of the innate antiviral immune response. Remarkably, a drop in the JEV titer occurred at 7 dpi and was associated with a temporary decrease of OAS1 and IFNβ mRNA expression. In our previous research using samples from the same pigs that are studied here, we showed that JEV replication in the CNS was efficiently suppressed by 21 dpi, in contrast to what is described here for the tonsils. This suppression in the CNS was associated with a strong activation of OAS1 expression. In those CNS tissues where the OAS response was not sufficient to control JEV replication, a strong induction of chemokine gene expression occurred, and was associated with a simultaneous extensively increased IFNγ expression and an extensive infiltration of T cells, which seemed to be the major limiting factor for JEV replication in this compartment (Redant et al., 2020). Although the increased IFNβ and OAS1 expression could not control JEV replication in the tonsils, no increase in innate chemokine mRNA expression such as CCL5 and CCL2 was observed, and thereby no immune cell infiltration will occur. Also, virtually no innate inflammatory immune response was observed in the tonsils, judging by the lack of upregulated mRNA of IL6, IL1a, TNFα, and components of the NLRP3 inflammasome. Of interest, a similar lack of inflammatory responses is also believed to contribute to the observation that, for instance, bats, alike pigs for JEV, represent a major reservoir for several viral pathogens. In those animals, a mechanism for downregulation of TNFα is observed as well as a lack of certain genes involved in inflammasome sensing. This lack of inflammatory immune response, which otherwise contributes to the suppression virus replication, would allow viruses to replicate to high levels while at the same time protecting the animals that serve as reservoir from inflammation-induced disease, in contrast to humans and other dead-end hosts suffering from severe immune-driven pathologies (Channappanavar and Perlman, 2017; O’Connor, 2018; Subudhi et al., 2019). Furthermore, only a slight raise in anti-inflammatory IL10 and FOXP3 mRNA was observed in the tonsils of JEV infected pigs at 10 dpi. Such a small increase alone may not suffice to suppress the inflammatory response. So other mechanisms that regulate inflammatory response might be involved and this should be further elucidated. In contrast to what was found in the CNS (Redant et al., 2020) and in line with the weak induction of the innate immune response, no raise in IFNγ mRNA expression could be observed throughout the entire *in vivo* experiment in the tonsils. Hence, this suggests that persistent JEV replication in the tonsils may be supported by the lack of an appropriate local IFNγ response. Indeed, in a mouse model of JEV, IFNγ secretions help to protect against lethal JEV by promoting viral clearance through T cells (Larena et al., 2013).

Combining all our observations, we hypothesize that the inadequate induction of the innate immune response and the absence of local IFNγ production in the tonsils may be an important factor in the inability of the porcine host to clear JEV efficiently from this site. The marked changes in T lymphocyte subpopulation frequencies in the tonsils of infected animals seems to further support this hypothesis. From the moment of detection of viral RNA in the tonsils (3 dpi), the frequency of CD4^+^CD8^+^ double-positive T cells significantly decreased with a concomitant significant decrease in CD8^+^, CD4^+^, and γδ T cells. Finally, by 21 dpi, the frequency of the CD8^+^ CD4^+^ cells was well below those observed at 1 and 2 dpi prior to virus detection. Importantly, the frequency of CD4^+^CD8^+^ double-positive T cells never restored completely to baseline levels during the entire experiment and remained significantly decreased till 21 dpi together with CD4^+^ single-positive T cells. Since the CD4^+^CD8^+^ double-positive cells are generally more abundant (around 25% of all lymphocytes in the tonsils), the decrease in this lymphocyte subtype outweighs the similarly observed decrease in CD4^+^ T cells or CD8^+^ T cells (both approximately 4% of all lymphocytes in the tonsils).

The role of other cell types such as NK cells and monocytes in the tonsils remains less clear. The NK cells in the tonsils already make up for a very small proportion of lymphocytes, making them difficult to study. Here, the frequency was found to decrease by 21 dpi; however, this was not significant. Lastly, when looking at the monocytic cell populations in the tonsils, there is a noticeable but not significant increase in frequency of the macrophage subtype. By 7 dpi, this population has tripled in frequency, and this coincides with the moment that JEV is present at a lower viral titer and a modest increase in CCL4 mRNA was observed. This might mean that these phagocytic cell types were recruited to the tonsils in response to the ongoing viral infection. By 21 dpi, the frequency of this immune cell subpopulation is again at steady-state levels.

Besides the insights that were obtained on JEV persistence and the induced immune response in tonsils, this study also highlights that JEV persists in the tonsils despite a potent induction of the humoral and cell-mediated immune response as measured in serum and PBMCs. The results from VNTs confirm previous reports indicating a potent induction of the antibody response with detection of JEV-specific neutralizing antibodies from 5 to 7 dpi onwards (Ricklin et al., 2016a; Ricklin et al., 2016b; Redant et al., 2020). Importantly, we present here for the first time clear indications that JEV infection in pigs not only induces a humoral immune response, but also induces a cell-mediated immune response upon restimulation of PBMCs with NS3 starting from 6 dpi. This had solely been studied before in humans and pointed that non-structural viral proteins such as NS3 are frequently targeted by T cells (Kumar et al., 2004; Turtle et al., 2016).

In pigs, CD4^+^CD8^+^ double-positive T cells represent a large proportion of T lymphocytes, which is in contrast to humans in whom their role in immunity against viruses has not been studied extensively. It is however known that these CD4^+^CD8^+^ double-positive T cells have properties of mature antigen experienced cells and that their proliferation and activity can be induced by stimulation with recall antigen, indicating that at least in pigs, this population contains memory/effector T cells (Zuckermann and Hussmann, 1996; Saalmüller et al., 1999). Indeed, when looking at the cell-mediated immunity upon restimulation with NS3, we found that the CD4^+^CD8^+^ double-positive T-cell population showed the strongest proliferative responses as well as the highest IFNγ production response, followed by the CD8^+^ T-cell population. Earlier research already pointed out that mainly the CD4^+^CD8^+^ double-positive T cells contribute to the IFNγ response among all subtypes of T cells in the pig (Rodriguez-Carreno et al., 2002). In addition, in other viral infections in pigs such as African swine fever virus (ASFV), it was demonstrated that UV-inactivated virus stimulated proliferation not only of CD4^+^ and CD8^+^ T cells but also of CD4^+^CD8^+^ double-positive T cells. Moreover, the stimulation of PBMCs with ASFV resulted in IFNγ produced by a population of CD4^+^CD8^+^ double-positive T cells (Takamatsua et al., 2013). Furthermore, in porcine reproductive and respiratory syndrome virus (PRRSV) infection, the CD4^+^CD8^+^ double-positive population plays an important role in recovery from PRRSV infection, which, in that case, appears to be mainly due to the cytotoxic function of this cell subtype (Chung et al., 2018). Similar to what was found in the tonsils, the frequency of CD4^+^CD8^+^ double-positive T cells was significantly decreased by 21 dpi in PBMCs. On the other hand, and in contrast to the situation in the tonsils, the CD8^+^ T-cell and CD4^+^ T-cell compartment did not decrease in the PBMCs until the end of the experiment.

NK cells also showed a strong IFNγ production upon restimulation with NS3 peptides. However, it was shown before that active depletion of NK cells did not result in a better outcome to JEV, so the impact of these cells in JEV pathogenesis might be rather low (O’Connor, 2018). Up to now, not much is known about γδ T cells in flavivirus infection. γδ T cells mainly function as a bridge between the innate and adaptive immunity, although it is known that these cells can also play in important role in memory function in bacterial infections and inflammation (Guerra-Maupome et al., 2019; Comeaua et al., 2020). Memory functions of γδ T cells have however only been recorded in viruses infecting primates. In the present study, proliferation of γδ T cells was also observed as a response to recall antigen, suggesting that they possibly may display a memory function in JEV infections in pigs. However, overall, this lymphocyte subtype does not seem to contribute as much in the defense against JEV infections, in particular, if compared to the more pronounced role that CD8^+^ and CD4^+^CD8^+^ double-positive subtypes seem to play.

Finally, when observing the role of monocytic subtypes upon JEV infection, no obvious changes could be observed in the cDC1 and cDC2 populations in the PBMCs, although the CD14^+^ monocyte population and CD172a^+^CD163^+^ populations first increase by 7 dpi and subsequently decrease by the end of the experiment. Although additional research would be needed to further investigate this, this might mean that, in pigs, JEV targets these types of cells as is also seen in ZIKAV patients where the CD14^+^ monocytes were significantly decreased (Michlmayr et al., 2017), and in DENV infections, it is also already known that monocytes are the main targets for virus replication, resulting in cell death (Castillo and UrcuquiInchima, 2018). When looking at these monocytic cell populations in the tonsils, there is a noticeable increase in frequency of the macrophage subtype. By 7 dpi, this population has tripled in frequency, and this coincides with the moment that JEV is present at a lower viral titer and a modest increase in CCL4 mRNA was observed. This might mean that these phagocytic cell types were recruited to the tonsils in response to the ongoing viral infection. By 21 dpi, the frequency of this immune cell subpopulation is again at steady-state levels.

In summary, our results indicate that JEV replication in tonsils induces a different local immune response than previously observed in CNS tissues. In tonsils, only a weak innate antiviral response is triggered, as indicated by a significant increase of only IFNβ and OAS1 mRNA expression. This antiviral response is, however, inadequate to halt JEV replication. Interestingly, no increase in expression of innate inflammatory cytokines or other inflammation associated factors were observed throughout the entire *in vivo* experiment in the tonsils. In addition, a complete lack of IFNγ mRNA expression was seen in the tonsils, which might further contribute to the persistence of JEV in the tonsils of infected pigs. This is furthermore associated with a decreased frequency of CD4^+^CD8^+^ double-positive cells in tonsils. Overall, this local suppression of anti-viral immunity probably favors a continuous production of virus progeny, which makes pigs such an efficient intermediate host for the JEV. Besides the insights provided on JEV replication, persistence, and induced immune responses in tonsils, our results clearly show that this persistence in the tonsils occurs despite a strong induction of the adaptive immune system whereby JEV-activated CD4^+^CD8^+^ T lymphocytes seem to play a prominent role.

## Data Availability Statement

The datasets supporting the conclusions of this article are included within the article and its additional files.

## Ethics Statement

The animal study was reviewed and approved by the ethical committee of Sciensano (20171024-01 and 20200602-01).

## Author Contributions

VR performed, designed, and optimized all experiments, analyzed the results, and wrote the manuscript. HF and KD provided advice and reagents. WC supervised the *in vivo* experiment. NR designed the *in vivo* experiment and reviewed/revised the manuscript. All authors contributed to the article and approved the submitted version.

## Funding

This study was funded by the Federal Public Service of Health, Food Chain Safety and Environment (RF17/6319 and RF18/6329), Belgium.

## Conflict of Interest

The authors declare that the research was conducted in the absence of any commercial or financial relationships that could be construed as a potential conflict of interest.

## Publisher’s Note

All claims expressed in this article are solely those of the authors and do not necessarily represent those of their affiliated organizations, or those of the publisher, the editors and the reviewers. Any product that may be evaluated in this article, or claim that may be made by its manufacturer, is not guaranteed or endorsed by the publisher.

## Supplementary Material

Supplementary Figure 1 Gating strategy for lymphocytes detection in the blood. Lymphocytes were gated according to their light scatter properties. Staining for CD3 is a first step in differentiating T cells from NK cells since T cells are characterized by the expression of CD3 and NK cells by the lack of CD3 expression. NK cells were then further gated on the expression of CD16 and CD8 and T cell subpopulations were discriminated by gating on γδ TCR or CD4 and CD8 expression. The same strategy was used for determining these subsets among tonsil lymphocytes.

Supplementary Figure 2 Detection of cell-mediated immune responses upon *in vitro* stimulation of PBMCs from non-infected animals with NS3 antigen. **(A)** no proliferation can be observed in CD4^+^CD8^+^ double-positive T cells, CD8^+^ T cells and γδ T cells **(B)** nor can any IFNγ production be observed in CD4^+^CD8^+^ double-positive T cells, CD8^+^ T cells and NK cells upon restimulation with NS3 antigen of PBMCs from non-infected animals.
