# Supplementary figures and images for: Japanese Encephalitis Virus Persistence in Porcine Tonsils Is Associated With a Weak Induction of the Innate Immune Response, an Absence of IFNγ mRNA Expression, and a Decreased Frequency of CD4+CD8+ Double-Positive T Cells
Source: Front Cell Infect Microbiol. 2022 Feb 24;12:834888. doi: 10.3389/fcimb.2022.834888 (PMC8908958; doi:10.3389/fcimb.2022.834888)

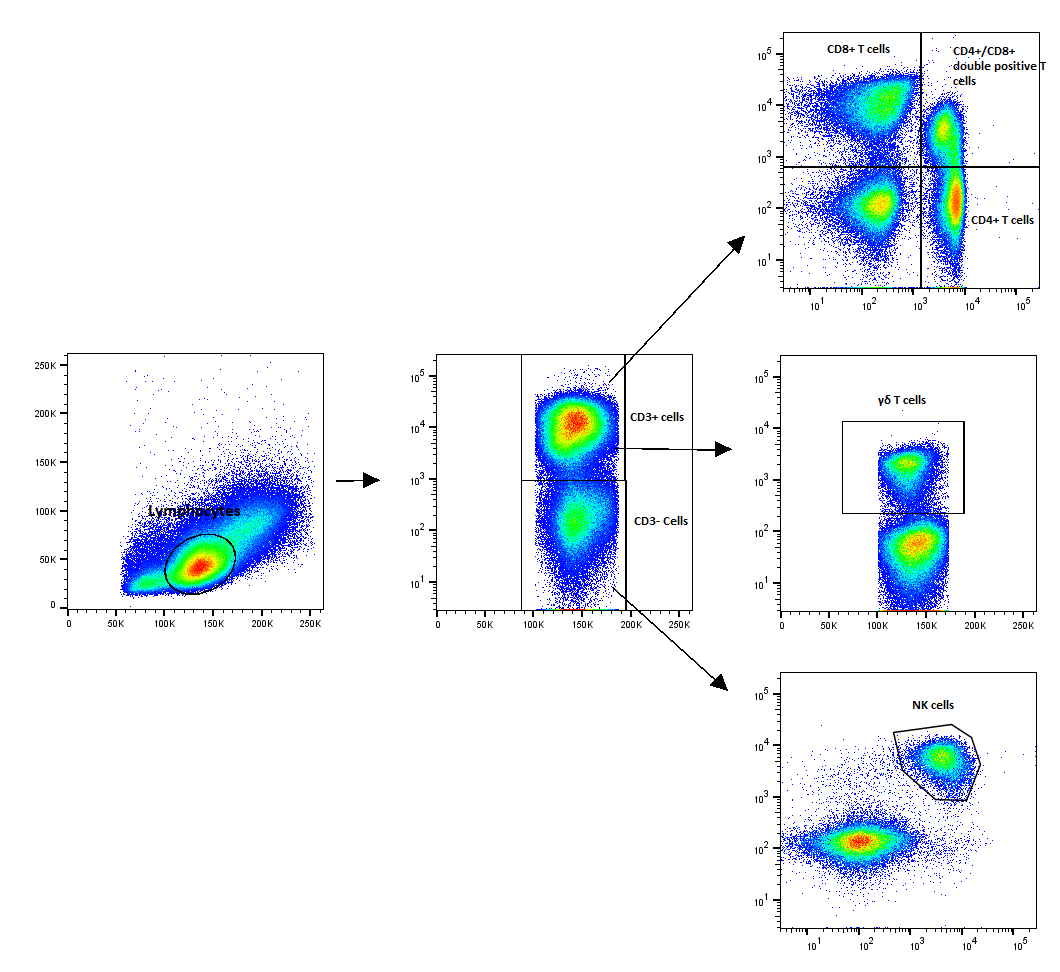

Supplement: Supplementary Figure 1 — Gating strategy for lymphocytes detection in the blood. Lymphocytes were gated according to their light scatter properties. Staining for CD3 is a first step in differentiating T cells from NK cells since T cells are characterized by the expression of CD3 and NK cells by the lack of CD3 expression. NK cells were then further gated on the expression of CD16 and CD8 and T cell subpopulations were discriminated by gating on γδ TCR or CD4 and CD8 expression. The same strategy was used for determining these subsets among tonsil lymphocytes. [file Image_1.tif]

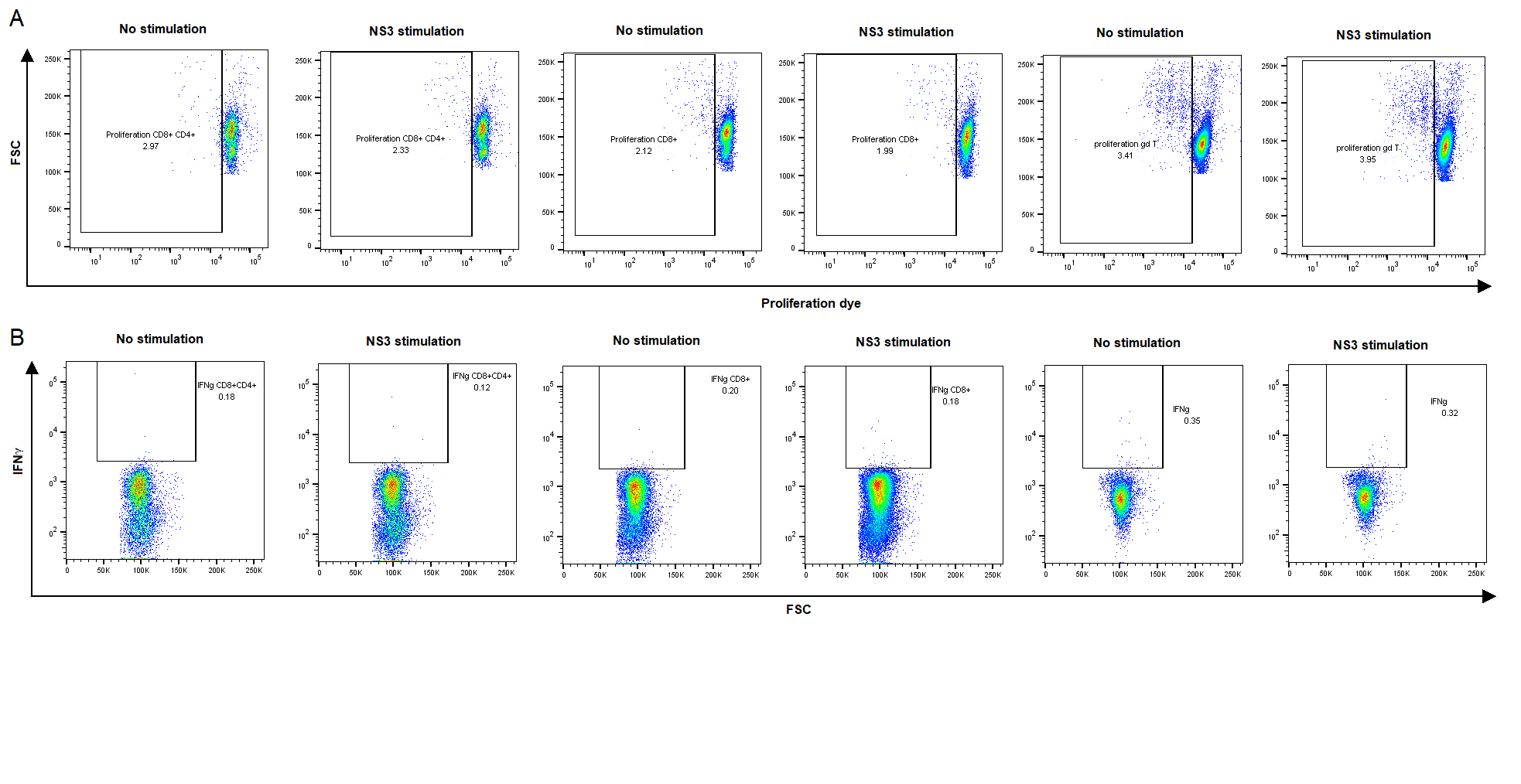

Supplement: Supplementary Figure 2 — Detection of cell-mediated immune responses upon in vitro stimulation of PBMCs from non-infected animals with NS3 antigen. (A) no proliferation can be observed in CD4+CD8+ double-positive T cells, CD8+ T cells and γδ T cells (B) nor can any IFNγ production be observed in CD4+CD8+ double-positive T cells, CD8+ T cells and NK cells upon restimulation with NS3 antigen of PBMCs from non-infected animals. [file Image_2.tif]
